# A New Tool for Probabilistic Assessment of MPS Data Associated with mtDNA Mixtures

**DOI:** 10.3390/genes15020194

**Published:** 2024-01-31

**Authors:** Jennifer A McElhoe, Alyssa Addesso, Brian Young, Mitchell M Holland

**Affiliations:** 1 Forensic Science Program, Department of Biochemistry & Molecular Biology, Pennsylvania State University, University Park, PA 16802, USA; alyssa.addesso@cox.net (A.A.); mmh20@psu.edu (M.M.H.); 2 NicheVision LLC, 526 South Main St., Akron, OH 44311, USA; brian@nichevision.com

**Keywords:** forensic biology, next-generation sequencing, massive parallel sequencing, mtDNA, mixture deconvolution, heteroplasmy

## Abstract

Mitochondrial (mt) DNA plays an important role in the fields of forensic and clinical genetics, molecular anthropology, and population genetics, with mixture interpretation being of particular interest in medical and forensic genetics. The high copy number, haploid state (only a single haplotype contributed per individual), high mutation rate, and well-known phylogeny of mtDNA, makes it an attractive marker for mixture deconvolution in damaged and low quantity samples of all types. Given the desire to deconvolute mtDNA mixtures, the goals of this study were to (1) create a new software, MixtureAceMT™, to deconvolute mtDNA mixtures by assessing and combining two existing software tools, MixtureAce™ and Mixemt, (2) create a dataset of in-silico MPS mixtures from whole mitogenome haplotypes representing a diverse set of population groups, and consisting of two and three contributors at different dilution ratios, and (3) since amplicon targeted sequencing is desirable, and is a commonly used approach in forensic laboratories, create biological mixture data associated with two amplification kits: PowerSeq™ Whole Genome Mito (Promega™, Madison, WI, USA) and Precision ID mtDNA Whole Genome Panel (Thermo Fisher Scientific by AB™, Waltham, MA, USA) to further validate the software for use in forensic laboratories. MixtureAceMT™ provides a user-friendly interface while reducing confounding features such as NUMTs and noise, reducing traditionally prohibitive processing times. The new software was able to detect the correct contributing haplogroups and closely estimate contributor proportions in sequencing data generated from small amplicons for mixtures with minor contributions of ≥5%. A challenge of mixture deconvolution using small amplicon sequencing is the potential generation of spurious haplogroups resulting from private mutations that differ from Phylotree. MixtureAceMT™ was able to resolve these additional haplogroups by including known haplotype/s in the evaluation. In addition, for some samples, the inclusion of known haplotypes was also able to resolve trace contributors (minor contribution 1–2%), which remain challenging to resolve even with deep sequencing.

## 1. Introduction

The field of forensic DNA is evolving rapidly as new technologies and methods become available and are validated. With increased sensitivity and throughput capabilities, mitochondrial (mt) DNA typing has become increasingly attractive, especially for forensic investigations involving samples with limited amounts of DNA [1], and including DNA mixtures [2]. The historic limitation of traditional mtDNA typing has been a lower discrimination potential created by a combination of uniparental inheritance of the mitochondrial (mito) genome and limited resolution of mixtures associated with Sanger sequencing. The increased sensitivity currently achievable with massively parallel sequencing (MPS) enables accurate characterization and reporting of intrinsic, low-level sequence mitogenome variants [3,4,5,6,7], and an advantage of uniparental inheritance is an expectation that the majority of individuals will only contribute one haplotype to a mixed sample making mtDNA an attractive aid in the assessment of mixed DNA samples [2,8,9,10].

DNA mixtures have always been a part of forensic casework and have increasingly become more common in forensic investigations [11,12]. The observed increase in mixed DNA samples is presumably due to rapidly advancing DNA technologies with increased sensitivity along with changes in sampling strategies and methodologies [11,13]. An example of this advancement is the ability of laboratories to generate profile information from trace amounts of DNA collected from touched objects. The increased resolution has resulted in approximately 15% of all evidence samples containing mixtures [14], and a retrospective study by Melton et al. [15] evaluating 691 casework hair samples indicated that ~9% of the samples were observed to have a mixture of mtDNA sequences, including heteroplasmic sequences. When samples with observations of mixed base calls are encountered, most forensic laboratories do not interpret the results and categorize the findings as inconclusive for reporting purposes [15]. The inability to fully identify and deconvolute mixtures is a recognized challenge when performing mtDNA analysis. As stated by the Spanish and Portuguese-Speaking Working Group of the ISFG (GHEP-ISFG) when performing a multi-laboratory study on mixture interpretation, “It is worth mentioning that mtDNA in mixture samples is not included in the evaluation of the Intercomparison Exercise program (IE). This is owed to the difficulties in interpreting mixtures from haplotype sequences from both technical and theoretical points of view.” [16]. This clearly illustrates that analysis of mtDNA mixtures is not a routine practice, and that work in this area is needed to provide the tools necessary to conduct such analyses. In order to retain the evidentiary value of these mixed samples, forensic laboratories need the ability to deconvolute mixed mtDNA sequences which will require software and bioinformatic solutions.

A presumed mtDNA mixture is typically not easily resolved, even with an MPS approach, as reconstructing complete haplotypes from short sequencing reads requires considerable effort and is not amenable to manual techniques. In addition, the sensitivity of MPS analysis and the ability to detect low-level mixtures (1–2%) increases the observation of complicating factors including heteroplasmy in one or more of the contributors and system-associated analytical noise. While some mixtures are relatively straightforward and easy to interpret, others are complex and can be challenging to deconvolute. The past several years has seen a rise in the application of probabilistic genotyping software to short tandem repeat analysis for the interpretation of mixtures. While strides have been made in this area allowing for the deconvolution of complex mixtures, there remains a proportion of cases that cannot be fully resolved. An overview by Coble and Bright [17], focusing on autosomal mixture interpretation using probabilistic approaches noted, “there is a strong need to apply probabilistic methods to interpreting mixtures with haploid marker systems”, such as mtDNA. To date, little work has been done to deconvolute mixtures of mtDNA sequence from MPS data [2,8,18,19]. The availability of MPS mtDNA data presents an opportunity to shift this paradigm.

The UNT Center for Human Identification in collaboration with the Tallinn University of Technology, and a team of researchers at the Universities of California in Santa Cruz and Davis collaborating with the Children’s Hospital Oakland Research Institute (CHORI), have taken the first steps towards development of software solutions. The first group applied phased MPS data for mixture deconvolution and were able to identify the major contributor in all mixtures but had difficulties in assessing point heteroplasmy (PHP) in some samples, making it difficult to determine whether the PHP should have been assigned to the major or minor contributor [18]. This approach showed promise but as noted by the authors, “mtDNA mixture analysis will become more effective and efficient with advances in bioinformatics pipelines”. UNT made subsequent advancements in developing pipelines using approaches focused on population and statistical phasing [8,9]. The mitochondrial mixture deconvolution and interpretation tool (MMDIT) is an open-source software that automates mixture deconvolution [8], but the shiny app is tailored to data generated using the Ion S5 and Converge software (ThermoFisher, Waltham, MA, USA) making it less user-friendly for data generated on an Illumina MiSeq or other sequencing platforms. The second group developed an advanced bioinformatic approach to deconvoluting complex mtDNA mixtures from MPS data using phylogenetic information [2]. The algorithm, Mixemt, is an opensource computer program written in Python and executed from a command line window and has been demonstrated to be effective in the deconvolution of mtDNA mixtures generated using shotgun sequencing data and a general-purpose DNA preparation kit. While the improved bioinformatic approach showed increased potential, the approach used shotgun sequencing as opposed to amplicon sequencing, the latter being more desirable and more commonly used in forensic laboratories. In addition, Mixemt utilizes a complex pipeline written in Python, requiring knowledge of command line coding. Clearly, a powerful bioinformatic approach is necessary for the deconvolution of mixtures, but in order for the approach to be widely adopted there is a need for the pipeline to be packaged in a user-friendly graphical user interface that does not require advance coding knowledge and that can use sequencing data from multiple platforms.

The principal objective of the current study was to assess a probabilistic approach to mitogenome mixture analysis that can be easily implemented as a tool in forensic laboratories. This was accomplished by combining Mixemt with a user-friendly software package, MixtureAce MT™ (NicheVision Forensics, LLC, Akron, OH, USA), to deconvolute mitochondrial mixtures using phylogenetic principles. Biological and in-silico mixtures were used to characterize the range and applicability of the new probabilistic approach.

## 2. Materials and Methods

### 2.1. In-Silico Mixtures

The initial evaluation of the Mixemt algorithm was accomplished using an in-silico dataset generated from small amplicon sequencing data. The in-silico dataset consisted of 126 two- (*n* = 63) and three-person (*n* = 63) mixtures created using previously sequenced (MiSeq) sole-source buccal swab samples enriched for mitogenome using the Promega PowerSeq™ Whole Mito System kit (Table S1). Sole-source samples were haplotyped using the revised Cambridge reference sequence (rCRS; [20,21]) and GeneMarker^®^ HTS (GM HTS; SoftGenetics, State College, PA, USA) and haplogroups were determined using Haplogrep2 [22]. Samples were selected to ensure a diverse cross section of different haplotype populations representing various similar and dissimilar haplotypes and included heteroplasmic (nine individuals with one site, three individuals with two sites, and 19 individuals with no sites of heteroplasmy; with a minor allele frequency (MAF) range of 6.4–47.0%) and homoplasmic contributors (Table S2). Considering the Expectation Maximization (EM) algorithm and Phylotree database used by the Mixemt software for mixture deconvolution, similarity was determined through the evaluation of haplogroup defining (diagnostic) single nucleotide polymorphisms (SNPs) and calculation of the number of SNPs shared between mixture contributors (percent shared haplogroup defining SNPs).

For the in-silico mixtures, pair-wise comparisons of the different haplotype combinations indicated that the range of percent shared haplogroup defining SNPs for similar haplotypes was 10–89.3% while the dissimilar haplotypes had a range of 0–7.7%. Once contributor samples were identified, a bioinformatic pipeline was used to create mixture files. Briefly, Samtools (v. 1.11; [23]) was used on GeneMarker^®^ HTS (GM HTS; SoftGenetics, State College, PA) generated alignment files (bam files) to subset and sort reads mapping to the rCRS for each sole-source contributor. Bamtools, v.2.5.2 [24], then converted each mapped and sorted bam file into two fastq files (read 1 & read 2) and seqtk (1.3-r116-dirty; https://github.com/lh3/seqtk.git was used to create randomly selected without replacement subsets of reads from each fastq file. Fastq subsets were combined in various ratios, 1:1, 1:3, 3:1 for two-person and 1:1:1, 1:3:3, 3:3:1 for three-person mixtures, with each mixture consisting of a total of 720 K and 840 K read fragments, respectively.

### 2.2. Biological Mixtures

To effectively assess the new combined software tool for deconvoluting mixtures, it was important to create a set of biological mixtures that were different from the in-silico mixtures used in the initial evaluation. Again, samples were selected to ensure a diverse cross section of different haplotype populations representing various similar and dissimilar haplotypes and included heteroplasmic (two individuals with one site, three individuals with two sites, and six individuals with no sites of heteroplasmy; with a MAF range of 2.4–41.1%) and homoplasmic contributors (Table S3). The biological dataset of 120 two- and three-person mixtures (Table S4) was generated using two amplification methods: the PowerSeq™ Whole Mito System (*n* = 60; Promega™ Corporation) and Precision ID mtDNA Whole Genome Panel (*n* = 60; Thermo Fisher Scientific by Applied Biosystems™,Waltham, MA, USA,). The PowerSeq™ System spans the entire mitogenome using a single multiplex of 161 overlapping amplicons (average length 167 bp), while the Precision ID Panel is composed of 162 amplicons (in two multiplexes) with an average amplicon length of 162 bp. The library preparation for the PowerSeq kit followed the manufacturer recommendations while the library preparation for the Precision ID kit was replaced with an Illumina ampliseq library preparation to allow for MiSeq sequencing. Biological mixtures were created using sole source buccal samples combined in various ratios and included a range of similar and dissimilar haplotype combinations (Table S2). Briefly, genomic DNA was isolated from buccal samples, mtDNA was quantified for each extract using a custom mtDNA qPCR assay [25], extracts were mixed together in various ratios (99:1, 49:1, 19:1, 9:1, and 1:1 for two-person mixtures; 3:3:1, 1:1:1, and 1:3:3 for three-person mixtures), and amplified using the two commercially available whole mtgenome amplification kits. All sequencing was conducted on a MiSeq instrument. Samples were batched in groups of 30 plus controls.

### 2.3. Creation of the User-Friendly Graphical User Interface: MixtureAce MT™

The principal objective was to develop software for the implementation of the EM method of Vohr et al. [2] in a user environment suitable for routine use by forensic laboratories and for PCR-targeted sequence data. Unlike Vohr, who implemented his method in Linux and specifically applied it to shotgun sequencing data, the new software runs in a cloud environment compatible with Windows platforms, and accommodates data from PCR-targeted sequencing, including the PowerSeq mtDNA kit and the Precision ID kit. In addition, the new software implements methods for reducing noise from sequencing errors and from sequences arising from potential nuclear mtDNA segments (NUMTs) or heteroplasmy.

MixtureAce MT™ software uses MPS generated sequencing files to determine the number of contributors and their respective mtDNA haplogroup in suspected mixed DNA samples. The software is designed to be easily used in a typical forensic laboratory environment with a user-friendly graphical user interface (Figure S1) allowing for the uploading of sequencing files, selection of parameter settings for analysis, and displaying results. Sequence files from suspected mixtures can be in a fastq or bam file format with the fastq format being required for application of noise filtration features.

Standard implementation of the MixtureAce MT™ software is cloud-based on a hosted Linux server due to the required Linux operating system and processing power requirements, which can be significant. The user interface on the cloud-based format runs as an application in a browser running on a local Windows computer. To date, the MixtureAce MT™ user interface has been validated on the following browsers: Chrome, Brave, and Microsoft Edge. An alternative implementation is also available and consists of running MixtureAce MT™ inside the Windows Subsystem for Linux (WSL) which is built into Windows versions 10 and later. In the alternative implementation, the browser-embedded user interface points to the WSL rather than to an external server. MixtureAce MT™ preprocesses the sequencing input file to reduce noise and expedite the deconvolution process. Preprocessing reduces false signals that may affect haplogroup calls and reduces confounding features such as NUMTs and noise through the targeting of sequencing error, primer-directed errors, and NUMT contamination.

Sequencing error is corrected in paired-end MPS sequencing files based on the proposition that when paired reads are generated from the same PCR amplicon and differ at a given nucleotide position (np) both reads cannot be correct. To accomplish error correction, the reverse read is reverse-complemented, orienting both reads in the forward direction relative to the reference sequence, the two reads are compared, and if the sequencer-generated base call at two or fewer nps differ between the two strands, then the base call with the higher sequencer-generated quality score is determined to be the correct base call and the lower-quality base is replaced with the correct base call [26].

Another type of error targeted by preprocessing is error resulting from the use of degenerative PCR primers in the amplification of target DNA, or primer-directed error. A PCR primer sequence is called degenerate if one or more of its nps have several possible bases [27]. Most PCR primer sets used by vendors of forensic DNA analysis kits contain a percentage of primers that are a mixture of primer sequences intended to increase the amplification efficiency for the target amplicon based on the known frequencies of polymorphic nps within the human mitogenome. The presence of a mixture guarantees that a certain percentage of the primers will be mismatched to the template and yet still be able to amplify at low levels as non-specific amplification products and be incorporated into the resultant amplicon. The incorporation of these mismatched primers into the amplicon can present as low-level variants or erroneous sequences (noise) that are not actually present in the DNA template sequence [28,29]. In addition, mtgenome amplification kits typically generate overlapping amplicons, and therefore amplicons can encompass the primer position of a neighboring primer pair, and some forensic kits, notably the PowerSeq kit, are known to incorporate PCR primers internally to the amplicon through run-on amplification, or amplicon concatenation mechanisms [30]. To reduce primer-directed error, known primer sequences were trimmed from sequencing reads when they appeared at one or both termini of reads.

Identification and filtering of potential NUMTs is accomplished via differential alignment analysis. Briefly, input sequencer reads are aligned to both the nuclear and the mitogenome and a Levenshtein distance is used to determine the difference between each read and the two different alignments. Levenshtein distance between two strings (i.e., strings of DNA sequence) is the number of insertions, deletions, and/or substitutions required to transform a source string (i.e., input sequencer read) into a target string (i.e., reference sequence string). The more different two strings of sequence are, the higher the Levenshtein number. More specifically, input sequencer reads are evaluated for similarity to the human nuclear genome to identify candidate NUMT reads [31]. Reads are aligned to the Hg38 reference genome (UCSC, December 2013 assembly of the human genome, GRCh38 Genome Reference Consortium Human Reference 38) using bowtie2 [32], a Levenshtein distance is calculated, and reads that have not been soft-clipped and have a Levenshtein distance less than three are sub-set as candidate NUMT reads. Next the candidate reads are mapped by the Burrows-Wheeler Aligner [33] to a “circularized” rCRS mitochondrial reference (circularization permits accurate alignment of amplicons across the control region) and the Levenshtein distance is again calculated. Reads are classified as NUMT reads and removed from the input sequencer reads if the Levenshtein distance to the nuclear reference is less than the Levenshtein distance to the mitochondrial reference, meaning the read has greater similarity to the nuclear genome sequence.

After preprocessing, contributor proportions and haplogrouping estimates are generated using the probabilistic technique of Vohr et al. [2]. In this method, both the contributor proportions and the haplogroup of each contributor is estimated simultaneously. The method finds the maximum likelihood estimate of both parameters using an expectation maximization procedure [34]. The method is phylogenetically directed in the sense that individual reads are assigned likelihoods of belonging to each individual haplogroup of the Phylotree build 17 [35]. The software identifies the best-supported haplogroups as those receiving the most support from read likelihoods and counts.

**Figure 1:**
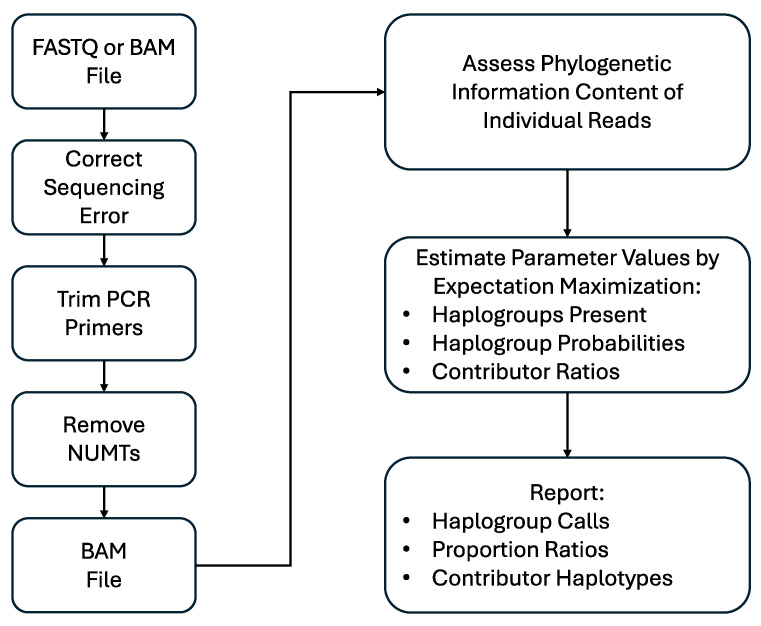
Schematic diagram of the MixtureAce MT™ pipeline.

MixtureAce MT™ reports the topmost supported haplogroups and the contribution proportion of the best-supported haplogroups. The haplogroup calls are substantiated by a listing of the phylogenetically diagnostic variant positions (diagnostic SNPs) that point to the haplogroup call and provide a partial haplotype from the haplogroup defining SNPs. A schematic of the MixtureAce MT™ pipeline is presented in Figure 1.

### 2.4. Statistical Analysis and Figures

Statistical analysis and figure generation was done in R [36] and RStudio [37]. Datasets were assessed using Shapiro-Wilks normality testing (stats package; Rv4.3.1) and found to have non-normal distributions. Subsequent testing employed non-parametric testing using Wilcoxon rank sum test (stats package; Rv4.3.1). Plotting was done in ggplot2_3.4.3 [38] and used cowplot_1.1.1 [39].

## 3. Results

### 3.1. In-Silico Mixtures

**Figure 2:**
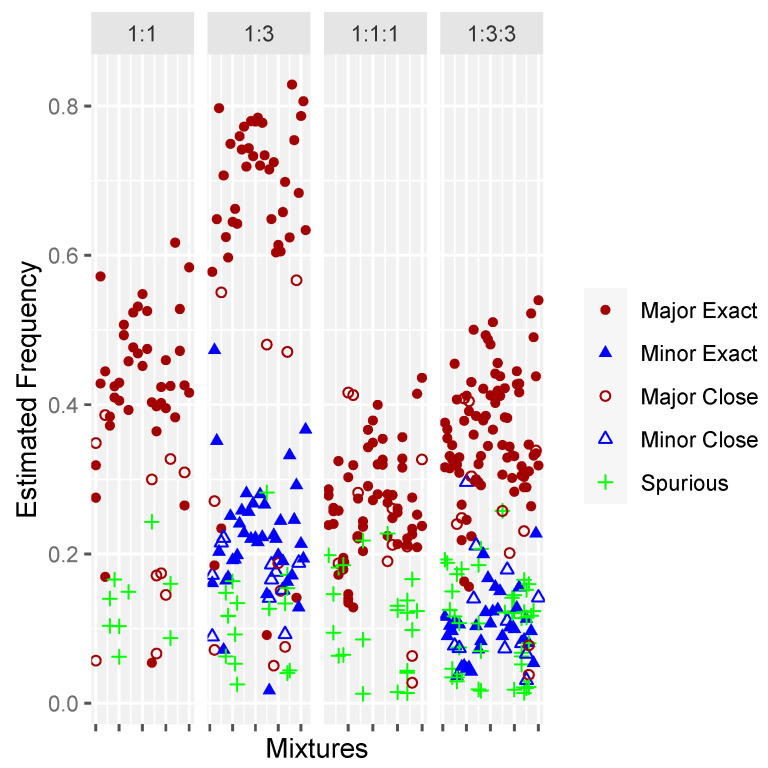
Frequency estimates for in-silico mixtures of 2- and 3-individuals at various dilution ratios. Filled circles and triangles represent contributors for which Mixemt identified the exact haplogroup and outlined circles and triangles represent identification of a closely related haplogroup. Samples that produced spurious haplogroups are represented by a “+”.

For the two- and three-person in-silico mixtures, Mixemt was able to detect the correct haplogroups for 95% of contributors and increased to 100% when closely related (i.e., R11 detected versus the exact haplogroup of R11a) haplogroups were included. Major and minor contributors were successfully identified in all mixtures at all dilution ratios (Figure 2).

**Figure 3:**
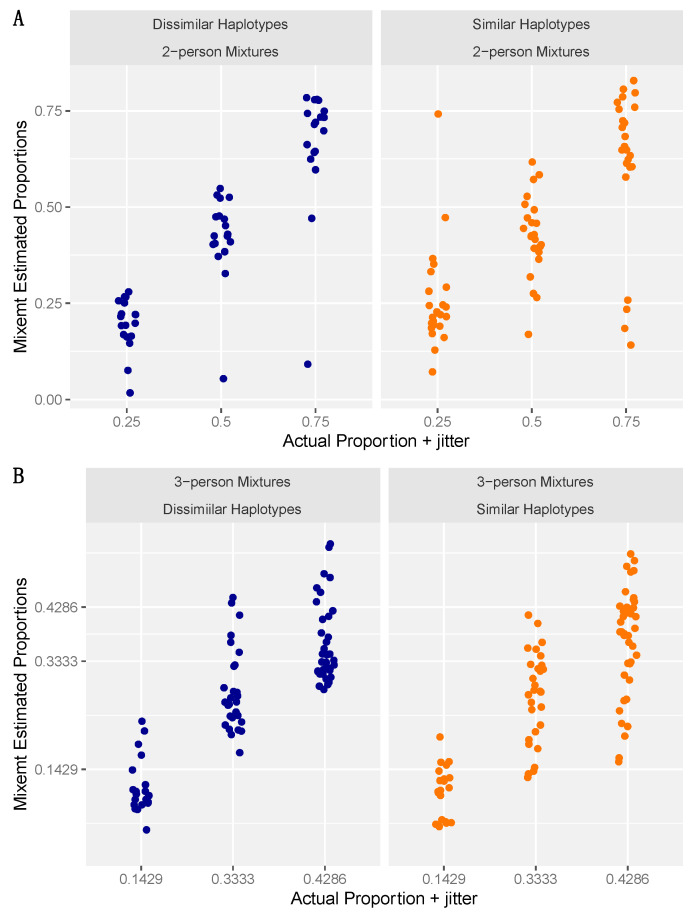
Summary results of Mixemt generated estimated haplogroup proportions and actual fragment proportions used to generate the mixture. (**A**) in-silico 2-person and (**B**) in-silico 3-person mixtures. The jitter function was applied for better visualization of overlapping datapoints.

To assess the accuracy of haplogroup contribution estimation by Mixemt in the in-silico mixtures the actual proportion of fragments subsetted to generate the mixtures was subtracted from the Mixemt estimated proportions. This resulted in negative values representing an underestimation and positive values expressing overestimation. Two-person mixtures with similar and dissimilar haplotypes were not found to be significantly different (*p* = 0.98) with a range of −0.61 to 0.22 and an average of −0.07 (7% under estimation) for similar haplotypes and a range of −0.65–0.048 and an average of −0.07 for dissimilar haplotypes. Three-person mixtures were also not significantly different (*p* = 0.37) and slightly more accurate on average with similar haplotypes ranging from −0.27 to 0.093 and an average of −0.055 and dissimilar mixtures ranging from −0.16 to 0.13 and averaging −0.050. Wilcoxon testing of the median values for the two- and three-person mixtures indicated that the difference was also insignificant (*p* = 0.99). Figure 3 presents the actual frequencies versus Mixemt estimated frequencies for all samples.

Spurious haplogroups were observed in the in-silico dataset with 25 spurious haplogroups identified in seven different pairs of individuals in the 2-person mixtures and 63 spurious haplogroups identified in 10 different sets of individuals in the 3-person mixtures. Due to the number of spurious haplogroups observed, analysis of the in-silico datasets was extended, reprocessing a subset of samples with spurious haplogroups in Mixemt and applying the known haplogroup function to include the contributor haplotypes in the deconvolution. Inclusion of known haplotypes resolved the spurious haplogroups.

### 3.2. Biological Mixtures

Biological mixtures were evaluated using GeneMarker HTS (GM HTS, SoftGenetics, State College, PA, USA) and the new combined tool, MixtureAce MT™. GM HTS analysis of sequencing files from the PowerSeq panel produced an average coverage of 10,345x with an average maximum of 43,212x and an average minimum coverage of 1204x, while the Precision ID kit produced an average coverage of 3858x with an average 17,916x maximum and 74.5x minimum coverage. The proportion of the mtgenome with coverage less than 2000x was 1.8% (average) for the the PowerSeq kit and 25.7% (average) for the Precision ID kit.

**Figure 4:**
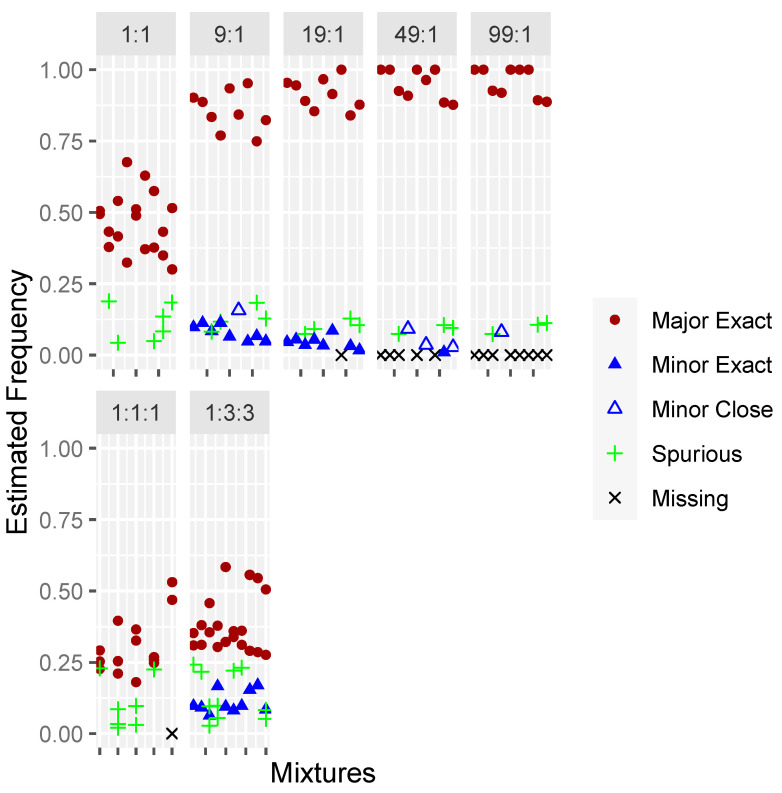
Frequency estimates for Precision ID amplified biological mixtures of 2- and 3- individuals at various dilution ratios. Filled circles and triangles represent contributors for which MixtureAce MT™ identified the exact haplogroup and outlined circles and triangles represent identification of a closely related haplogroup. Contributors not detected by the software are represented by an “x” and samples that produced spurious haplogroups are represented by a “+”.

Evaluation of the Precision ID dataset showed that MixtureAce MT™ was able to detect the correct haplogroups for 95% of contributors in samples with minor contribution ≥ 5%, and that the minor contributor failed to be detected in 72% of mixtures with minor frequencies of 1–2% (Figure 4).

Spurious haplogroups were observed in the Precision ID mixtures with 7 spurious haplogroups identified in two different pairs of individuals in the 2-person mixtures and 11 spurious haplogroups identified in four different sets of individuals in the 3-person mixtures. Extended testing of the inclusion of known contributors was conducted on the Precision ID dataset, using 28 mixtures that produced spurious haplogroups and reprocessing those samples with the inclusion of two known contributors. When two known contributor haplotypes were included in the deconvolution, all spurious haplogroups were resolved and produced exact match (*n* = 65) or close match (*n* = 3) for major and minor contributors. The inclusion of known haplotype also increased the resolution of trace contributors present in all seven of the samples that had dropout of the minor contributor at 2% and in 1/8 samples with dropout at 1% minor contributor.

**Figure 5:**
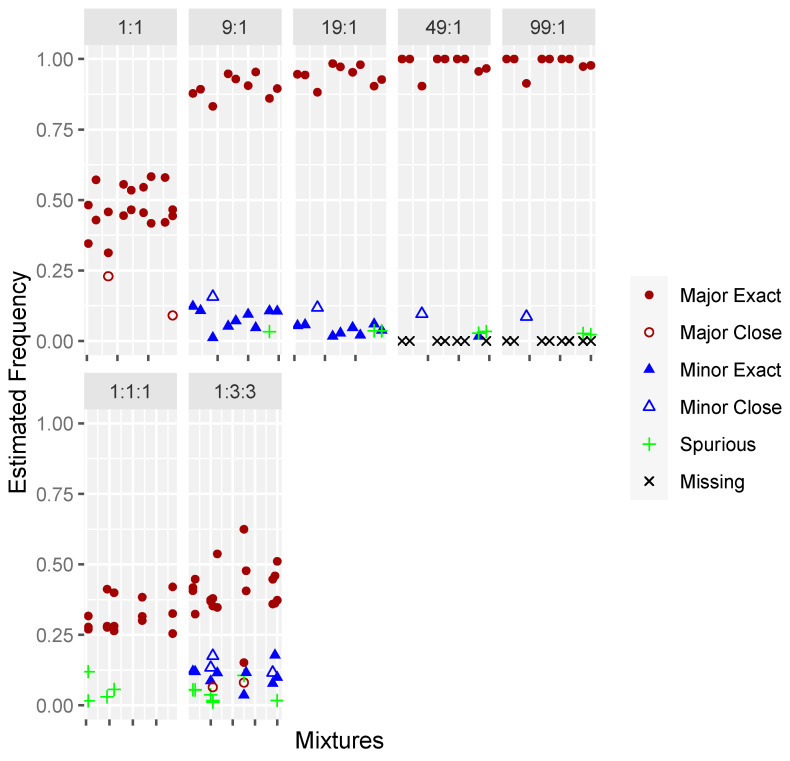
Frequency estimates for PowerSeq WGM amplified biological mixtures of 2- and 3- individuals at various dilution ratios. Filled circles and triangles represent contributors for which MixtureAce MT™ identified the exact haplogroup and outlined circles and triangles represent identification of a closely related haplogroup. Contributors not detected by the software are represented by an “x” and samples that produced spurious haplogroups are represented by a “+”.

Evaluation of the PowerSeq dataset showed that MixtureAce MT™ was able to detect the correct haplogroups for 100% of contributors in samples with minor contribution ≥ 5%. The minor contributor failed to be detected in 83% of mixtures with minor frequencies of 1–2% (Figure 5).

Spurious haplogroups were again observed in the PowerSeq WGM dataset with 18 spurious haplogroups identified in the same two pairs of individuals in the 2-person mixtures and 11 spurious haplogroups identified in the same 10 sets of individuals in the 3-person mixtures that were observed in the Precision ID dataset. Since the same spurious haplogroups were observed in the same samples as the Precision ID samples set, an evaluation of the inclusion of known haplotypes was not conducted on the PowerSeq mixtures.

**Figure 6:**
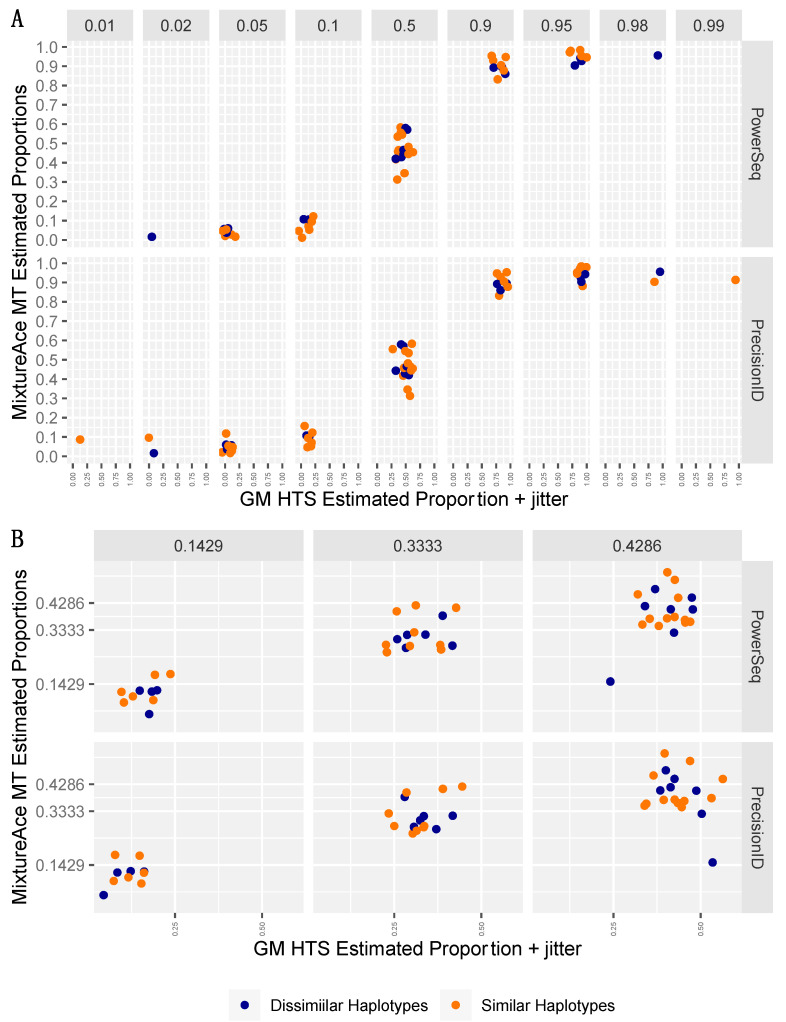
Summary results of GeneMarker estimated biological mixture proportions versus MixtureAce MT™ estimated proportions for (**A**) 2-person and (**B**) 3-person mixtures amplified using the PowerSeq and Precision ID amplification kits. Note: the PowerSeq 2-person dataset failed to resolve 1% minor contributors and therefore the 0.99 and 0.01 plots have no datapoints. The jitter function was applied for better visualization of overlapping datapoints.

To assess the accuracy of MixtureAce MT™ to estimate the haplogroup contributions in the biological mixtures, an independent assessment of the contributor proportions was generated using GM HTS and subtracted from the MixtureAce MT™ estimated proportions for mixture ratios ≥ 5%. This resulted in negative values representing an underestimation and positive values expressing overestimation. The accuracy the Precision ID two-person mixtures with similar haplotypes ranged from −0.25 to 0.21 with an average of −0.010 (1% under estimation) while mixtures with dissimilar haplotypes ranged from −0.15–0.15 and had an average of −0.010. The proportion estimates for the PowerSeq dataset were less accurate and tended to overestimate proportions for two-person mixtures with similar (−0.11 to 0.32, avg. 0.04) and dissimilar (−0.012 to 0.09, avg. 0.04) haplotypes. A similar result was produced for the three-person mixtures with similar (−0.12 to 0.10, avg. −0.017) and dissimilar (−0.31 to 0.22, avg. −0.034) haplotypes in the Precision ID dataset and similar (−0.097 to 0.089, avg. −0.018) and dissimilar (−0.17 to 0.068, avg. −0.026) haplotypes in the PowerSeq dataset. Wilcoxon testing indicated a statistically significant difference within the PowerSeq dataset when comparing two-person and three-person mixtures (*p* = 0.00013), but did not indicate a significant difference when comparing two-person and three-person mixtures in the Precision ID dataset (*p* = 0.29) or two-person similar and dissimilar haplotypes (Precision ID *p* = 0.73; PowerSeq *p* = 0.55), or 3-person similar and dissimilar haplotypes (Precision ID *p* = 0.79, PowerSeq *p* = 0.86). Comparison between the Precision ID and PowerSeq datasets also showed no significant difference (*p* = 0.071). Figure 6 presents the GM HTS estimated proportions versus MixtureAce MT™ estimated frequencies for all samples.

### 3.3. Preprocessing

**Figure 7:**
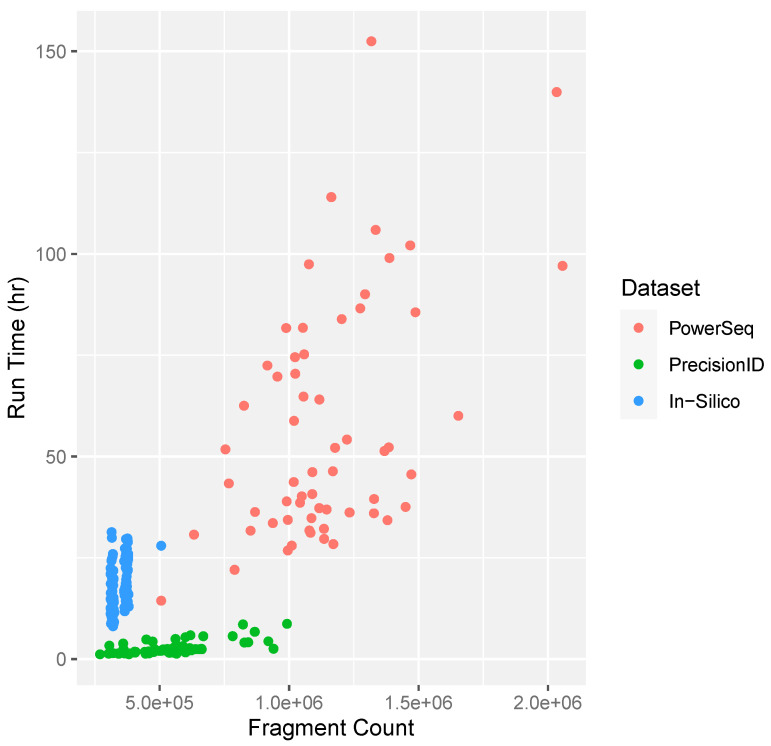
Mixture deconvolution run time based on the sequencing file size represented as the number of sequencing fragments used for haplogroup and frequency estimation. Pink: PowerSeq datasets, no pre-processing. Green: Precision ID datasets preprocessed with MixtureAce MT^TM^. Blue: in-silico datasets, no preprocessing.

One of the major drawbacks of the previously available software for mtDNA mixture deconvolution has been that implementation can be time-consuming [10]. MixtureAce MT™ reduces confounding features such as potential NUMTs and noise which reduces the time required for the EM algorithm and heuristic filters to produce haplogroup and frequency estimates. Figure 7 highlights the general positive correlation between fragment count and time, the influence of dissimilar and similar haplotypes within the in-silico dataset, as well as the decrease in run time for Precision ID samples that were preprocessed with MixtureAce MT™ and the PowerSeq dataset without preprocessing.

A summary of the preprocessing metrics for the Precision ID mixture dataset including input reads, read correction run time, primmer trimming run time, alignment run time, number of possible NUMTs identified, the run times for NUMT identification & removal, the number of NUMTs ultimately removed, and the total time required for preprocessing is provided in Table 1.

### 3.4. Random Reduction

To further investigate the time requirements for the deconvolution process and to assess depth of coverage requirements, a subset of preprocessed biological mixture samples (*n* = 14) was run with an initial average number of fragments of 800 K (mtgenome coverage of ~10 K) that were randomly reduced to various fragment counts ranging from 1.5 K to 20 K fragments per mixture. The sample subset contained similar and dissimilar haplotypes in two- and three-person mixtures at various ratios. Identifiers for the biological mixtures are structured as: contributor 1:contributor 2 and contain sample number & major haplogroup clade for each contributor with the mixture ratio given in parentheses. All mixtures tested except for 5U:10U (19:1), 7D:5U (49:1) and 4D:7D (99:1) produced correct haplogroups for the initial reduction to 20 K fragments (~215x mtgenome coverage; Table 2). For the trace contributors at 49:1 and 99:1 the read fragments were further increased to 35 K and 50 K but still failed to identify the minor contributors. The correct identification of exact major and exact minor contributors was observed in eight of the remaining eleven samples when reduced to 10 K fragments, four samples (plus one sample with an exact major and a closely related minor haplogroup reported) at 3 K, and two samples were still producing the correct contributors when reduced to a total of 1500 read fragments per sample. Some of the mixtures continued to produce spurious haplogroups similar to the additional haplogroups reported for samples with the full complement of fragments.

## 4. Discussion

In this study, Mixemt was able to correctly identify the haplogroups for 95–100% of contributors in both in-silico and biological mixtures, and successfully identified the major and minor contributors in all in-silico mixture ratios and 95% of biological mixtures with minor contribution ≥ 5%. The accuracy of Mixemt to estimate haplogroup proportions closely followed the actual mixture proportions and, on average, were slightly less than the true contribution. The observations in this study on haplogroup identification and estimated proportions are similar to the findings by Vohr in the original Mixemt study, with the underestimation of proportions most likely due to the software not redistributing a portion of the fragments assigned to closely related and/or low frequency haplogroups during the evaluation of all haplogroups [2]. Other observations in the present work differ from the Vohr study with an increased incidence of spurious or additional haplogroups and an increased level of minor dropout at higher fragment depths.

The number of spurious haplogroups in both the in-silico and the biological datasets in this study were higher than what Vohr observed. This could be due to differences in a shotgun versus an amplicon sequencing approach, the differences in the number of fragments in each mixture, or differences in Mixemt settings, but is most likely due to the present study having a small amplicon size, a high number of fragments per sample, differences in contributor haplotypes and mixture combinations, and multiple contributors having haplotype differences (private mutations) from Phylotree haplogroup defining polymorphisms. Due to a low density of variants in the coding region, private mutations in the coding region are unlikely to overlap any other variant site within the same fragment, and the small size of fragments (~200 bps) generated by our sequencing protocol also reduce the chance that multiple variants will be located on the same fragment. In this study, all of the additional haplogroups belonged to the H subclade of Phylotree and resulted from the identification of 1 or 2 diagnostic variants. For example, in-silico mixture 787 (B2o), 832 (L1c2b1a’b) had additional haplogroups H3aq (1018A) and H27/H27e (11719A and 16129A) for each of the three ratios (1:1, 1:3, and 3:1). All three of these variants are contained in the haplotype of individual 832, are not associated with the haplogroup L1c2b1a’b, but are diagnostic for H3aq and H27/H27e. The Vohr study noted spurious H subclade haplogroups and saw an increase in additional haplogroups when the number of sample fragments (10 K, 20 K, and 40 K) was increased. While Vohr observed spurious H haplogroups when one of the true haplogroups (true: H1 [2]) in a mixture was closely related to the additional haplogroup (spurious H1s and H26), this was only observed for three out of eleven 3-person in-silico combinations in our dataset. Ultimately, all of the spurious haplogroups in our datasets were resolved by adding known haplotypes during deconvolution. The addition of known haplotypes also led to resolution of the minor contributor in all of the 49:1 and in one 99:1 biological mixtures. Inclusion of known haplotype (profile) information in mixture deconvolution is a common practice in forensic cases as persons of interest are commonly known or suspected to be contributors in DNA mixtures.

The biological datasets in this study had an average of 500,000 and 1 million reads for the Precision ID and PowerSeq amplifications, respectively. With an average fragment length of 200 bps for both amplification kits, and considering 5% minor contribution, this relates to approximately 300- and 600-fold coverage, and approximately 60- and 120-fold coverage for a 1% minor contribution across the mtgenome. At this high number of fragments, for both amplification kits, the major and minor contributors were correctly identified for 5% minor contribution but only one out of 15 mixtures (10U:7D; 49:1) with a minor contribution of 1–2% was resolved. The Vohr study found that in-silico samples consisting of 3000 fragments of approximately 300 bps (~3 fold coverage for 5% minor contribution) often failed to detect contributors present at less than 10% and increasing mixtures to 10,000 fragments could resolve minor contributors at 5% (our calculation: 9-fold coverage), but required 20,000–40,000 fragments to recover 1% (our calculation: 4- to 7-fold coverage) contribution in 20 and 31 out of 50 mixtures, respectively. The reason for the differences is unclear but could be due to differences in sequencing approaches and average fragment length or differences in contributor haplotypes and combinations.

### Heteroplasmy and Haplotype Reconstruction

The in-silico dataset contains nine individuals with one site, three individuals with two sites, and 19 individuals with no sites of heteroplasmy with MAFs ranging from 6.4 to 47.0%. Across all mixtures in the in-silico dataset, there was one trio of individuals (399:886:918) that had a spurious haplogroup that was possibly due to the 16093C heteroplasmic site (MAF 25.9%) in individual 886. A spurious haplogroup, H27 [1] was reported for each of the mixture ratios for this combination of individuals. The 16093C diagnostic SNP was observed with an estimated proportion of 12.4% in the 1:1:1 mixture, 11.7% in the 1:3:3 mixture, and 12.4% in the 3:3:1 mixture. No other indications of complications due to heteroplasmy were found in the in-silico or biological datasets. The biological dataset contains two individuals with one site, three individuals with two sites, and six individuals with no sites of heteroplasmy with a MAF range of 2.4–41.1%.

Haplotype reconstruction was attempted for a small subset of samples using the MixtureAce MT™ reported diagnostic variants. The proportion of the haplotype that was able to be reconstructed ranged from ~12% for minor contributors in mixtures with highly similar haplotypes to ~65% for major contributors in mixtures with similar haplotypes and both contributors in mixtures with dissimilar haplotypes. While complete reconstruction of an individual’s haplotype is not reported by MixtureAce MT™, the reported haplogroup along with the estimated proportions (unequal proportions required) enable most samples to be fully reconstructed through manual investigation of the sequencing pileup in an alignment software.

## 5. Conclusions

Overall, MixtureAce MT™ effectively detects the correct contributing haplogroups and closely estimates contributor proportions in sequencing data generated from small amplicons for mixtures with minor contribution ≥ 5%. The new software provides a user-friendly interface and reduces confounding features such as NUMTs and noise, reducing traditionally prohibitive processing times. Based on this study, processing times can be further diminished through random reduction of samples to 20 K fragments while maintaining the ability to successfully identify contributor haplogroups and reducing the time to run a sample by approximately 60%.

A challenge of mixture deconvolution using small amplicon sequencing is the generation of spurious haplogroups resulting from private mutations that differ from Phylotree but these additional haplogroups can be resolved by including known haplotype/s in the evaluation. The inclusion of known haplotypes can also help to resolve trace contributors (minor contribution 1–2%), which remain challenging to resolve even with deep sequencing. The outcomes of this work will help to increase the effectiveness of forensic mtDNA analysis, decrease the time and cost of data analysis compared to current standard practices, and allow for separation of components of a mitogenome mixture.

## Figures and Tables

**Table 1:** Summary MixtureAce MT™ preprocessing data for Precision ID biological mixtures.

	Input FastQ Reads	Read Correction Time (min)	Primer Trimming Time (min)	Circular Alignment Time (min)	NUMT Candidates	NUMT ID Time (min)	NUMT’s Removed	NUMT Removal Time (min)	Total Run Time (min)
**Average**	387,591	9.68	12.33	4.4	60,195	2.84	3915	2.42	31.66
**Maximum**	621,856	18.4	25.7	11.7	100,402	5.7	8114	5.3	64.20
**Minimum**	230,247	5.5	6.2	2.7	28,445	1.8	885	1.1	17.60

**Table 2:** Minimum number of reduced fragments per biological mixture sample required for the correct identification of major and minor contributors by MixtureAce MT^TM^. Biological mixture identifiers given as contributor 1 sample number major haplogroup clade:contributor 2 sample number major haplogroup clade (mixture ratio contributor 1:contributor 2). Coverage (cov) is the corresponding average depth of coverage across the mtgenome for the given number of fragments.

Minor Dropout	Minimum Number Fragments for Correct Major/Minor			
**20K** (~215x cov)	**20K** (~215x cov)	**10K** (~100x cov)	**3K** (~32x cov)	**1.5K** (~16x cov)
5U:10U (19:1)	4D:7D (19:1)	6J:8J (19:1)	4D:7D (1:1)	1J:6J (1:1)
7D:5U (49:1)	10U:2K:5U (1:3:3)	10U:7D (9:1)	10U:9A (9:1)	9A:10U:4D (1:1:1)
4D:7D (99:1)	10U:3K:2K (1:3:3)	10U:9A (19:1)	9A:11A (9:1) major exact minor close	
**Average runtime based on the number of fragments**				
Full complement	20K	10K	3K	1.5K
3.2 h	1.2 h	0.6 h	0.2 h	0.1 h

## Data Availability

Dataset available on request from the authors.

## Supplementary Materials

The following are available online at https://www.mdpi.com/article/10.3390/genes15020194/s1, Table S1: List of in-silico mixtures. (A) two person mixtures; (B) three person mixtures. Both tables list contributor ID, contributor haplogroup, the number of unique and shared haplogroup defining SNPs, and our determination of whether the mixture was considered to consist of similar or dissimilar haplotypes, Table S2: List of in-silico mixture contributors with haplogrep2 generated haplogroup and GM HTS generated haplotype and heteroplasmy information. Heteroplasmy is presented as rCRS nucleotide-nucleotide position-alternative nucleotide- frequency (%), Table S3: List of biological mixture contributors with haplogrep2 generated haplogroup and GM HTS generated haplotype and heteroplasmy information. Heteroplasmy is presented as rCRS nucleotide-nucleotide position-alternative nucleotide- frequency (%), Table S4: List of biological mixtures. (A) two-person mixtures; (B) three-person mixtures. Both tables list contributor ID, contributor haplogroup, the number of unique and shared haplogroup defining SNPs, and our determination of whether the mixture was considered to contain similar or dissimilar haplotypes. Figure S1: Screen shot images of the MixtureAce MT™ user-friendly interface. (A) Sample identification and run status, (B) Data type selection, (C) Data file upload, (D) Advanced options, (E) Contributor detection options, (F) Deconvoluting mtDNA mixtures by expectation maximization.
