# Supplementary material for: A New Tool for Probabilistic Assessment of MPS Data Associated with mtDNA Mixtures
Source: Genes (Basel). 2024 Jan 31;15(2):194. doi: 10.3390/genes15020194 (PMC10887502; doi:10.3390/genes15020194)
Supplement: Supplementary file 1 [file genes-15-00194-s001.zip › genes-2811925-supplementary.pdf]

## Supplemental Tables

**Table S1.** *List of in-silico mixtures. A.) two person mixtures; B.) three person mixtures. Both tables list contributor ID, contributor haplogroup, the number of unique and shared haplogroup defining SNPs, and our determination of whether the mixture was considered to consist of similar or dissimilar haplotypes.*

**A.)**

| Contributor 1 | Contributor 1 Haplogroup (Haplogrep2) | Contributor 2 | Contributor 2 Haplogroup (Haplogrep2) | Unique Haplogroup Defining SNPs | Shared Haplogroup Defining SNPs | Percent Shared Haplogroup Defining SNPs | Similar (S) or Dissimilar (D) Haplotypes |
|---|---|---|---|---|---|---|---|
| 20 | K1a1b2a1a | 235 | L1c1d1 | 58 | 3 | 4.92 | D |
| 20 | K1a1b2a1a | 832 | L1c2b1a'b | 64 | 3 | 4.48 | D |
| 27 | X2+225+@153 | 445 | 2b+226+16192 | 4 | 9 | 69.23 | S |
| 28 | M7c1c2 | 776 | Z4 | 26 | 4 | 13.33 | S |
| 45 | D1 | 1219 | D6a1a | 15 | 7 | 31.82 | S |
| 51 | J1c2 | 433 | T2 | 25 | 4 | 13.79 | S |
| 51 | J1c2 | 77 | T1a1 | 29 | 4 | 12.12 | S |
| 57 | G2a1c1 | 885 | D1 | 20 | 5 | 20.00 | S |
| 97 | V7 | 585 | H7a1 | 9 | 0 | 0.00 | D |
| 97 | V7 | 296 | H1a3 | 9 | 1 | 10.00 | S |
| 97 | V7 | 489 | H3g1 | 11 | 0 | 0.00 | D |
| 97 | V7 | 614 | H4a1a1a | 15 | 0 | 0.00 | D |
| 97 | V7 | 516 | H4a1a1a2 | 16 | 0 | 0.00 | D |
| 97 | V7 | 1355 | R11 | 17 | 0 | 0.00 | D |
| 234 | L2a1a2 | 307 | L2a1a2a1a | 3 | 25 | 89.29 | S |
| 234 | L2a1a2 | 471 | L2a1c3b1 | 12 | 18 | 60.00 | S |
| 307 | L2a1a2a1a | 471 | L2a1c3b1 | 16 | 18 | 52.94 | S |
| 351 | B2r | 1282 | D1 | 34 | 0 | 0.00 | D |
| 433 | T2 | 484 | J1c3 | 23 | 5 | 17.86 | S |
| 787 | B2o | 832 | L1c2b1a'b | 53 | 1 | 1.85 | D |
| 399 | A2g1 | 918 | A2+64+16129 | 6 | 15 | 71.43 | S |

**B.)**

| Contributor 1 | Contributor 1 Haplogroup (Haplogrep2) | Contributor 2 | Contributor 2 Haplogroup (Haplogrep2) | Contributor 3 | Contributor 3 Haplogroup (Haplogrep2) | Unique Haplogroup Defining SNPs | Shared Haplogroup Defining SNPs | Percent Shared Across All Contributors Haplogroup Defining SNPs | Similar (S) or Dissimilar (D) Haplotypes |
|---|---|---|---|---|---|---|---|---|---|
| 20 | K1a1b2a1a | 787 | B | 832 | L1c2b1a'b | 76 | 4 | 5.00 | D |
| 20 | K1a1b2a1a | 235 | L1c1d1 | 471 | L2a1c3b1 | 73 | 9 | 10.98 | S |
| 27 | X2+225+@153 | 445 | X2b+226+16192 | 489 | H3g1 | 9 | 9 | 50.00 | S |
| 27 | X2+225+@153 | 343 | A | 489 | H3g1 | 25 | 2 | 7.41 | D |
| 27 | X2+225+@153 | 97 | V7 | 471 | L2a1c3b1 | 35 | 2 | 5.41 | D |
| 45 | D1 | 1219 | D6a1a | 1282 | D1 | 10 | 12 | 54.55 | S |
| 45 | D1 | 57 | G2a1c1 | 1282 | D1 | 16 | 12 | 42.86 | S |
| 45 | D1 | 235 | L1c1d1 | 787 | B2o | 60 | 2 | 3.23 | D |
| 51 | J1c2 | 433 | T2 | 484 | J1c3 | 16 | 14 | 46.67 | S |
| 51 | J1c2 | 77 | T1a1 | 484 | J1c3 | 20 | 14 | 41.18 | S |
| 51 | J1c2 | 77 | T1a1 | 433 | T2 | 22 | 14 | 38.89 | S |
| 57 | G2a1c1 | 1219 | D6a1a | 1282 | D1 | 25 | 9 | 26.47 | S |
| 65 | L | 235 | L1c1d1 | 787 | B2o | 64 | 4 | 5.88 | D |
| 97 | V7 | 296 | H1a3 | 585 | H7a1 | 12 | 1 | 7.69 | D |
| 97 | V7 | 489 | H3g1 | 585 | H7a1 | 14 | 0 | 0.00 | D |
| 97 | V7 | 489 | H3g1 | 614 | H4a1a1a | 18 | 1 | 5.26 | D |
| 97 | V7 | 343 | A | 885 | D1 | 31 | 1 | 3.13 | D |
| 97 | V7 | 516 | H | 1355 | R11 | 26 | 1 | 3.70 | D |
| 234 | L2a1a2 | 307 | L2a1a2a1a | 471 | L | 8 | 25 | 75.76 | S |
| 235 | L1c1d1 | 787 | B2o | 832 | L | 44 | 25 | 36.23 | S |
| 399 | A2g1 | 886 | A2+64+16129 | 918 | A2ap | 7 | 15 | 68.18 | S |

**Table S2**. *List of in-silico mixture contributors with haplogrep2 generated haplogroup and GM HTS generated haplotype and heteroplasmy information. Heteroplasmy is presented as rCRS nucleotide-nucleotide position-alternative nucleotide- frequency (%).*

| In-silico Mixture Contributors | Haplogroup | Haplotype | Heteroplasmy |
|---|---|---|---|
| 20 | K1a1b2a1a | 73G,114T,152C,263G,315.1C,497T,750G,1189C,1438G,1811G,2706G,3480G,3777C,4769G,7028T,7278C,7729G,8860G,9055A,9698C,9800C,10046C,10398G,10550G,11299C,11467G,11719A,11914A,12308G,12372A,13326C,14167T,14766T,14798C,15326G,15758G,15924G,16093C,16224C,16311C,16519C | T16093T 16.26 |
| 27 | X2+225+@153 | 73G,195C,225A,263G,315.1C,750G,1438G,1719A,1888A,2706G,2772T,3483A,4769G,6221C,6371T,7028T,8860G,8966C,9438A,11293G,11719A,12705T,13966G,14470C,14766T,15326G,16182C,16183C,16189C,16223T,16248T,16278T,16519C | A153G 18.79<br>T11254C 19.72 |
| 45 | D1 | 73G,196C,263G,315.1C,489C,729C,750G,1438G,2092T,2706G,3010A,3918A,4769G,4883T,5178A,7028T,8414T,8701G,8860G,9443C,9540C,9739T,10097G,10398G,10400T,10822G,10873C,11410C,11719A,12705T,14668T,14766T,14783C,15043A,15301A,15326G,16223T,16325C,16362C | none |
| 51 | J1c2 | 73G,185A,188G,228A,263G,295T,315.1C,462T,489C,750G,1438G,2706G,3010A,4216C,4769G,7028T,8860G,10398G,11251G,11719A,12612G,13708A,14766T,14798C,15326G,15452A,16069T,16126C | T152C 29.76 |
| 57 | G2a1c1 | 73G,263G,309.1C,315.1C,489C,709A,750G,1438G,2706G,4769G,4833G,5021C,5108C,5601T,7028T,7600A,8701G,8860G,9377G,9540C,9575A,10398G,10400T,10873C,11719A,12705T,13563G,13780G,14200C,14569A,14766T,14783C,15043A,15301A,15326G,16093C,16183C,16189C,16194C,16195C,16223T,16227G,16278T,16362C,16519C | none |
| 65 | I2 | 73G,152C,199C,204C,207A,250C,263G,309.1C,315.1C,750G,1438G,1719A,2706G,4529T,4561C,4769G,7028T,8251A,8860G,10034C,10238C,10398G,11353C,11719A,12501A,12705T,13780G,14290C,14766T,15043A,15326G,15758G,15924G,16129A,16223T,16391A,16519C | none |
| 77 | T1a1 | 73G,152C,195C,263G,309.1C,309.2C,315.1C,524.1A,524.2C,709A,750G,1438G,1888A,2706G,4216C,4769G,4917G,7028T,8697A,8860G,9899C,10463C,11251G,11719A,12633A,13368A,14766T,14905A,15326G,15452A,15607G,15928A,16126C,16163G,16186T,16189C,16294T,16519C | none |
| 97 | V7 | 72A,93G,263G,309.1C,315.1C,750G,1438G,2706G,4580A,4769G,5054A,7028T,7444A,8860G,15326G,15904T,16298C | none |
| 234 | L2a1a2 | 73G,146C,152C,195C,263G,309.1C,309.2C,315.1C,750G,769A,1018A,1438G,2416C,2706G,2789T,3594T,3918A,4048A,4104G,4769G,5285G,7028T,7175C,7256T,7274T,7521A,7771G,8206A,8701G,8860G,9221G,9540C,10115C,10398G,10873C,11719A,11914A,11944C,12693G,12705T,13590A,13650T,14566G,14766T,15244G,15301A,15326G,15629C,15784C,16223T,16278T,16286T,16294T,16309G,16390A,16519C | T8908C 10.16<br>C16286C 6.39 |
| 235 | L1c1d1 | 73G,151T,152C,182T,186A,189C,195C,198T,247A,263G,297G,309.1C,315.1C,316A,523del,524del,750G,769A,825A,1018A,1438G,2395del,2706G,2758A,2885C,3594T,3666A,3796T,3843G,4104G,4500C,4769G,5108C,5460A,5951G,6071C,7028T,7055G,7146G,7256T,7389C,7521A,7948T,8027A,8468T,8655T,8701G,8860G,9072G,9540C,10321C,10398G,10586A,10688A,10810C,10873C,11719A,11899C,11914A,11984C,12705T,12810G,13105G,13485G,13506T,13650T,13789C,14000A,14148G,14178C,14560A,14766T,14911T,15301A,15326G,15626T,16038G,16108T,16129A,16187T,16189C,16223T,16278T,16284G,16293G,16294T,16311C,16360T,16519C | C16108C 35.84 |
| 296 | H1a3 | 56.1C,73G,263G,309.1C,309.2C,315.1C,750G,1438G,3010A,4769G,7444A,8860G,11242T,14560A,15326G,15757G,16051G,16162G,16288C | T16288T 23.84<br>T16519C 20.12 |
| 307 | L2a1a2a1a | 73G,146C,152C,195C,263G,315.1C,750G,769A,1018A,1438G,2416C,2706G,2789T,3594T,3918A,4104G,4769G,5285G,7028T,7175C,7256T,7274T,7521A,7771G,8206A,8701G,8860G,9211T,9221G,9540C,10115C,10398G,10454C,10873C,11719A,11914A,11944C,12693G,12705T,13590A,13650T,14566G,14766T,15211T,15244G,15301A,15326G,15421G,15629C,15784C,16092C,16223T,16278T,16286T,16294T,16309G,16390A,16519C | C16286C 11.83 |
| 343 | A2 | 73G,146C,152C,153G,235G,263G,315.1C,523del,524del,663G,750G,1438G,1736G,2706G,4248C,4769G,4824G,7028T,8027A,8794T,8860G,11009C,11719A,12007A,12705T,14766T,15032T,15326G,16111T,16223T,16290T,16319A,16362C,16519C | none |
| 351 | B2r | 73G,152C,189G,200G,263G,309.1C,315.1C,499A,750G,827G,960.1C,1438G,2706G,2833G,3547G,4769G,4820A,4977C,5492C,5899del,6473T,6752G,7028T,8281del,8282del,8283del,8284del,8285del,8286del,8287del,8288del,8289del,8860G,9950C,11177T,11719A,12372A,12904G,13590A,14374C,14766T,15326G,15535T,16183C,16189C,16217C,16278T,16456A,16519C | none |
| 399 | A2g1 | 64T,73G,146C,153G,235G,263G,309.1C,315.1C,523del,524del,663G,750G,1438G,1736G,2706G,4248C,4769G,4824G,4970G,7028T,7724T,8027A,8794T,8860G,11719A,12007A,12705T,13855T,14766T,15326G,16111T,16223T,16290T,16319A,16362C,16391A | none |
| 433 | T2 | 73G,146C,263G,309.1C,315.1C,709A,750G,1438G,1888A,2706G,4216C,4769G,4917G,7028T,8697A,8860G,10463C,11251G,11719A,11812G,13368A,14233G,14766T,14905A,15326G,15452A,15607G,15928A,16126C,16294T,16296T,16519C | T152C 11.5 |
| 445 | X2b+22+16192 | 73G,153G,195C,225A,226C,263G,524.1A,524.2C,750G,1438G,1719A,2706G,4769G,6221C,6371T,7028T,7052G,8393T,8860G,11719A,12705T,13198A,13708A,13966G,14470C,14766T,15326G,15927A,16189C,16192T,16223T,16265G,16278T,16519C | 16265A 47.02 |

| | | | |
|---|---|---|---|
| 471 | L2a1c3b1 | 73G,146C,195C,263G,309.1C,315.1C,750G,769A,1018A,1438G,2416C,2706G,2789T,3010A,3594T,4104G,4769G,6663G,7028T,7175C,7256T,7274T,7521A,7771G,8206A,8701G,8860G,9221G,9540C,9824C,9932A,10115C,10398G,10410C,10873C,11719A,11914A,11944C,12693G,12705T,13203G,13590A,13650T,14566G,14766T,15301A,15326G,15784C,15924G,16093C,16148T,16223T,16278T,16294T,16355T,16390A,16519C | none |
| 484 | J1c3 | 73G,185A,263G,295T,309.1C,315.1C,462T,489C,750G,1438G,2706G,3010A,4216C,4769G,5530T,7028T,7855T,8860G,10398G,11251G,11719A,12063T,12612G,13708A,13934T,14766T,14798C,15326G,15452A,16069T,16126C | none |
| 489 | H3g1 | 152C,263G,315.1C,750G,1438G,3992T,4418C,4769G,6776C,7967T,8860G,10235C,10754C,15326G,16223T,16325C,16519C | none |
| 516 | H4a1a1a2 | 73G,228T,263G,309.1C,315.1C,523del,524del,750G,1438G,3992T,4024G,4688C,4769G,5004C,5618C,8185C,8269A,8348G,8860G,9123A,10044G,11173T,13545T,14325C,14365T,14582G,15326G | none |
| 585 | H7a1 | 263G,309.1C,315.1C,750G,1438G,1719A,4769G,4793G,8860G,12063T,15326G,16261T,16519C | T152C 9.16 |
| 614 | H4a1a1a | 73G,263G,309.1C,315.1C,523del,524del,750G,1438G,3992T,4024G,4769G,5004C,8269A,8860G,9123A,10044G,14365T,14582G,15326G | A215G 29.56 |
| 787 | B2o | 73G,159C,263G,315.1C,499A,750G,827G,1438G,2706G,2804G,3547G,4769G,4820A,4977C,6473T,6647C,7028T,8281del,8282del,8283del,8284del,8285del,8286del,8287del,8288del,8289del,8292A,8860G,9950C,11177T,11719A,13590A,13879A,14766T,15326G,15535T,16092C,16182C,16183C,16189C,16217C,16519C | none |
| 832 | L1c2b1a'b | 73G,150T,151T,152C,182T,186A,189C,195C,198T,247A,263G,297G,315.1C,316A,750G,769A,825A,1018A,1438G,2220G,2395del,2706G,2758A,2885C,3591A,3594T,3666A,4104G,4769G,5495C,5899.1C,5951G,6071C,6150A,6253C,7028T,7055G,7064C,7076G,7146G,7256T,7337A,7389C,7521A,8027A,8468T,8655T,8701G,8784G,8860G,8877C,9072G,9540C,9861C,10031C,10321C,10398G,10586A,10589A,10688A,10792G,10793T,10810C,10873C,11164G,11654G,11719A,12049T,12477C,12705T,12810G,13105G,13149G,13485G,13506T,13650T,13789C,14000A,14178C,14560A,14766T,14911T,15067C,15326G,16129A,16166G,16187T,16189C,16223T,16230G,16265C,16278T,16284G,16286A,16294T,16311C,16519C,16527T | none |
| 885 | D1 | 73G,263G,315.1C,489C,750G,1438G,2092T,2706G,3010A,3816G,4659A,4769G,4883T,5178A,7028T,8414T,8701G,8860G,9540C,10398G,10400T,10873C,11719A,12705T,12771A,13320T,14668T,14766T,14783C,15043A,15217A,15301A,15326G,16223T,16325C,16362C | none |
| 886 | A2+(64)+16129 | 64T,73G,146C,153G,235G,263G,309.1C,315.1C,499A,523del,524del,663G,750G,1438G,1736G,2706G,4248C,4310G,4769G,4824G,6249A,7028T,8027A,8794T,8860G,10045C,10373A,11719A,12007A,12705T,14766T,15326G,16111T,16129A,16223T,16290T,16319A,16354T,16362C,16519C | T16093C 25.94 |
| 918 | A2ap | 64T,73G,146C,153G,235G,263G,309.2CC,315.1C,523del,524del,663G,750G,1438G,1736G,2706G,4248C,4769G,4824G,7028T,8027A,8794T,8860G,9809G,11719A,12007A,12705T,14766T,15326G,16111T,16189C,16223T,16290T,16319A,16362C | none |
| 1219 | D6a1a | 73G,263G,315.1C,489C,709A,750G,1438G,1719A,2706G,3714G,4769G,4883T,5178A,6701G,7028T,7424G,7879G,8020A,8618C,8701G,8860G,8895C,9540C,10398G,10400T,10873C,11719A,12654G,12705T,13194A,14766T,14783C,15043A,15301A,15326G,15553A,16182C,16183C,16189C,16223T,16274A,16362C | none |
| 1282 | D1 | 5C,61T,73G,131C,146C,263G,309.1C,309.2C,315.1C,489C,524.1A,524.2C,750G,1438G,2092T,2706G,3010A,4769G,4883T,5178A,7028T,8414T,8775T,8860G,9540C,10398G,10400T,10873C,11719A,11830C,12705T,14668T,14766T,14783C,15043A,15301A,15326G,15924G,16223T,16325C,16362C | none |
| 1355 | R11 | 73G,185A,189G,263G,309.1C,315.1C,709A,750G,1438G,2706G,4706G,4769G,5186C,5301G,6338G,7028T,8860G,9071T,10031C,10398G,10978G,11061T,11719A,12358G,12950G,13681G,14696G,14766T,15326G,16183C,16189C,16311C,16519C | none |

**Table S3.** *List of biological mixture contributors with haplogrep2 generated haplogroup and GM HTS generated haplotype and heteroplasmy information. Heteroplasmy is presented as rCRS nucleotide-nucleotide position-alternative nucleotide- frequency (%).*

| Biological Mixtures Contributor ID | Haplogroup | Haplotype | Heteroplasmy |
|---|---|---|---|
| 1J | J1c8a | A73G,G185A,G228A,A263G,C295T,C315insC,C462T,T489C,C524insAC,A750G,A1438G,A2706G,G3010A,T4216C,A4769G,C7028T,A8860G,T10084C,A11251G,G11719A,A12612G,G13708A,C14766T,T14798C,A15326G,C15452A,C16069T,T16126C,G16319A,T16368C | none |
| 2K | K1a1b2a1a | A73G,T152C,A263G,C315insC,C497T,C524insAC,A750G,C770T,T1189C,A1438G,A1811G,A2706G,A3480G,T3777C,A4769G,C7028T,T7278C,A7729G,A8860G,G9055A,T9698C,T9800C,A10398G,A10550G,A11053G,T11299C,A11467G,G11719A,G11914A,A12308G,G12372A,T13326C,C14167T,C14766T,T14798C,A15326G,A15758G,A15924G,T16093C,T16224C,T16311C,T16519C | 16093 11.77 |
| 3K | K2a | A73G,T146C,T152C,A263G,C315insC,G709A,A750G,A1438G,A1811G,A2706G,A3480G,T4561C,A4769G,C7028T,A8860G,G9055A,T9698C,T9716C,A10550G,T11299C,A11467G,G11719A,A12308G,G12372A,C14167T,C14766T,T14798C,A15326G,T16224C,T16311C,T16519C | 234G 7.53<br>7050G 34.5 |
| 4D | D4e2 | A73G,A263G,C315insC,T489C,A523del,C524del,A750G,A1438G,A2706G,G3010A,A4769G,C4883T,C5178A,C7028T,C8414T,A8701G,A8860G,T9540C,A10398G,C10400T,T10873C,C11215T,G11719A,C12705T,C14668T,C14766T,T14783C,G15043A,G15301A,A15326G,A15874G,C16223T,T16362C | none |
| 5U | U5a1a1 | A73G,A263G,C309insC,C315insC,A750G,A1438G,T1700C,A2706G,T3197C,A4769G,T5495C,T6351C,C7028T,A8860G,G9477A,A11467G,G11719A,A12308G,G12372A,T13617C,C14766T,A14793G,A15218G,A15326G,A15924G,C16256T,C16270T,A16399G | 251A 3.23<br>6351T 41.12 |
| 6J | J1c2c2 | A73G,T146C,G185A,A188G,G228A,A263G,C295T,C315insC,C462T,T489C,A750G,A1438G,A2706G,G3010A,T4216C,A4769G,C7028T,A8860G,G8865A,A10398G,G10685A,A11251G,G11719A,A12612G,T13281C,G13708A,A13933G,C14766T,T14798C,A15326G,C15452A,C16069T,T16126C,T16519C | 16286T 15.0 |
| 7D | D4b2a1 | A73G,A263G,C280T,C315insC,T489C,A523del,C524del,A750G,A1382C,A1438G,C1662T,A2706G,G3010A,A4769G,C4883T,C5178A,C7028T,G8020A,C8414T,A8701G,A8860G,C8964T,T9540C,T9824A,A10398G,C10400T,T10873C,G11719A,C12705T,G13708A,C14668T,C14766T,T14783C,G15043A,G15301A,A15326G,A15524G,C16223T,C16355T,T16362C | 200G 4.66<br>4883C 2.42 |
| 8J | J1c4 | A73G,G185A,A263G,C295T,C315insC,C462T,T489C,A750G,A1438G,A2706G,G3010A,T4216C,A4769G,A6503G,C7028T,A7271G,A8860G,A9632G,A10398G,A11251G,G11719A,T12083G,A12612G,T13641C,G13708A,C14766T,T14798C,A15326G,C15452A,C16069T,T16126C | none |
| 9A | A2ac1 | A73G,T146C,A153G,A235G,A263G,C309insC,C315insC,A523del,C524del,A663G,A750G,A1438G,A1736G,A2706G,T3083C,T4248C,A4769G,A4824G,C7028T,G8027A,C8794T,A8860G,G9055A,A9377G,A9473C,G11719A,G12007A,C12705T,A14687G,C14766T,T15074C,A15326G,C16111T,G16213A,C16223T,C16290T,G16319A,T16362C | none |
| 10U | U5a1a2a1 | A73G,A263G,C309insC,C315insC,C573insCC,A750G,A1438G,T1700C,A2706G,T3197C,A4769G,A5319G,T6293C,A6629G,T6719C,C7028T,A8860G,G9477A,T11152C,A11467G,G11719A,A12308G,C12346T,G12372A,T13617C,C14766T,A14793G,A15218G,A15326G,C16256T,C16270T,A16399G | none |
| 11A | A2k1a | C64T,A73G,T146C,A153G,A235G,A263G,C309insC,C315insC,A523del,C524del,A663G,A750G,A1438G,A1736G,A2706G,T3202C,T4248C,A4769G,A4824G,C7028T,G8027A,A8460G,C8794T,A8860G,G11719A,G12007A,C12705T,C14766T,A15326G,A15924G,C16111T,C16223T,C16290T,G16319A,T16362C | none |

**Table S4.** *List of biological mixtures. A.) two-person mixtures; B.) three-person mixtures. Both tables list contributor ID, contributor haplogroup, the number of unique and shared haplogroup defining SNPs, and our determination of whether the mixture was considered to contain similar or dissimilar haplotypes.*

**A.)**

| Contributor 1 | Contributor 1 Haplogroup (Haplogrep2) | Contributor 2 | Contributor 2 Haplogroup (Haplogrep2) | Unique Haplogroup Defining SNPs | Shared Haplogroup Defining SNPs | Percent Shared Haplogroup Defining SNPs | Similar (S) or Dissimilar (D) Haplotypes |
|---|---|---|---|---|---|---|---|
| 4D | D4e2 | 7D | D4b2a1 | 10 | 10 | 50.00 | S |
| 5U | U5a1a1 | 10U | U5a1a2a1 | 10 | 12 | 54.55 | S |
| 1J | J1c8a | 6J | J1c2c2 | 11 | 14 | 56.00 | S |
| 6J | J1c2c2 | 8J | J1c4 | 11 | 14 | 56.00 | S |
| 1J | J1c8a | 8J | J1c4 | 9 | 13 | 59.09 | S |
| 9A | A2ac1 | 11A | A2k1a | 10 | 14 | 58.33 | S |
| 9A | A2ac1 | 10U | U5a1a2a1 | 39 | 0 | 0.00 | D |
| 5U | U5a1a1 | 7D | D4b2a1 | 32 | 0 | 0.00 | D |
| 7D | D4b2a1 | 10U | U5a1a2a1 | 38 | 0 | 0.00 | D |

**B.)**

| Contributor 1 | Contributor 1 Haplogroup (Haplogrep2) | Contributor 2 | Contributor 2 Haplogroup (Haplogrep2) | Contributor 3 | Contributor 3 Haplogroup (Haplogrep2) | Unique Haplogroup Defining SNPs | Shared Haplogroup Defining SNPs | Percent Shared Across All Contributors Haplogroup Defining SNPs | Similar (S) or Dissimilar (D) Haplotypes |
|---|---|---|---|---|---|---|---|---|---|
| 3K | K2a | 10U | U5a1a2a1 | 2K | K1a1b2a1a | 32 | 14 | 30.43 | S |
| 10U | U5a1a2a1 | 2K | K1a1b2a1a | 5U | U5a1a1 | 30 | 13 | 30.23 | S |
| 1J | J1c8a | 6J | J1c2c2 | 8J | J1c4 | 12 | 15 | 55.56 | S |
| 4D | D4e2 | 5U | U5a1a1 | 9A | A2ac1 | 45 | 1 | 2.17 | D |
| 9A | A2ac1 | 10U | U5a1a2a1 | 4D | D4e2 | 50 | 1 | 1.96 | D |

**Figure S1.** *Screen shot images of the MixtureAce MT™ user-friendly interface.* ***A.*** *Sample identification and run status,* ***B.*** *Data type selection,* ***C.*** *Data file upload,* ***D.*** *Advanced options,* ***E.*** *Contributor detection options.*

**A.**

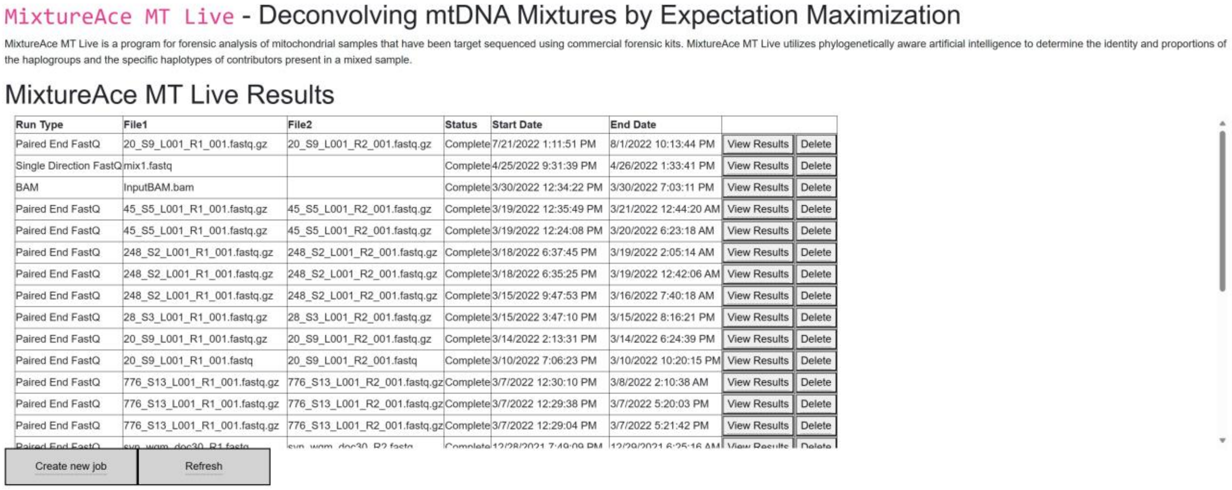

B.

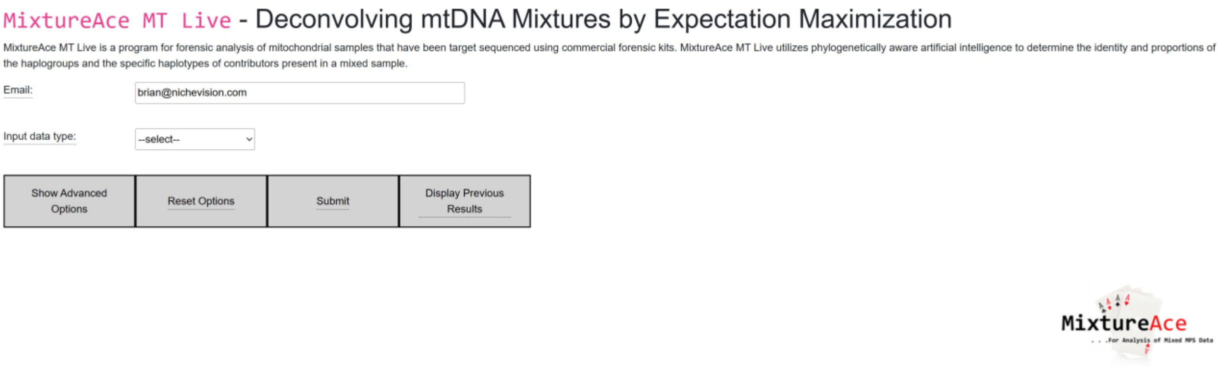
MixtureAce MT Live - Deconvolving mtDNA Mixtures by Expectation Maximization
MixtureAce MT Live is a program for forensic analysis of mitochondrial samples that have been target sequenced using commercial forensic kits. MixtureAce MT Live utilizes phylogenetically aware artificial intelligence to determine the identity and proportions of the haplogroups and the specific haplotypes of contributors present in a mixed sample.
Email:
brian@nichevision.com
Input data type:
--select--
Show Advanced Options
Reset Options
Submit
Display Previous Results
MixtureAce
For Analysis of Mixed MPS Data

C.

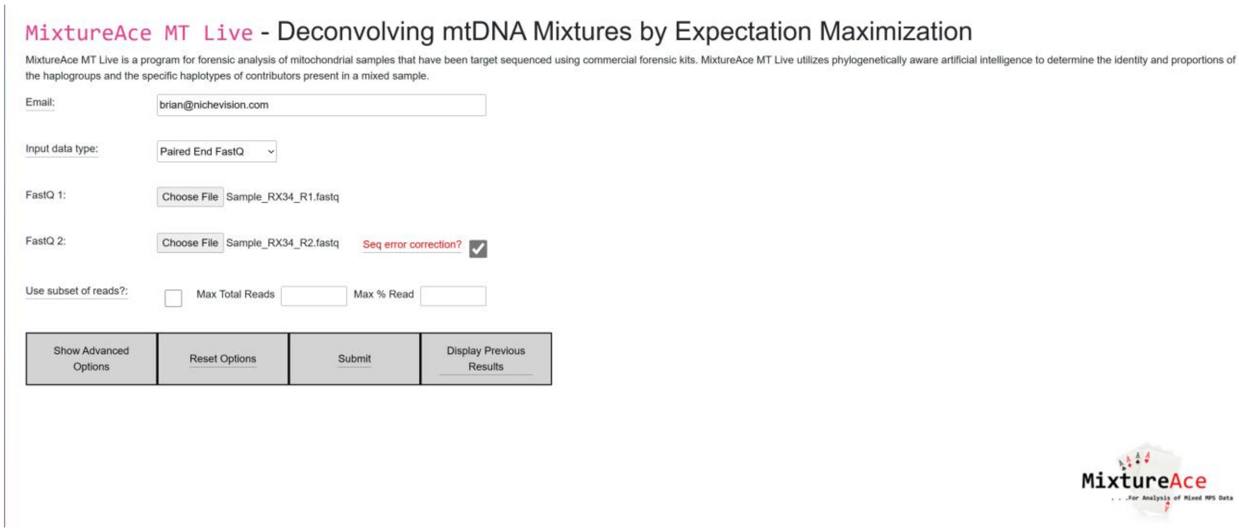
MixtureAce MT Live - Deconvolving mtDNA Mixtures by Expectation Maximization
MixtureAce MT Live is a program for forensic analysis of mitochondrial samples that have been target sequenced using commercial forensic kits. MixtureAce MT Live utilizes phylogenetically aware artificial intelligence to determine the identity and proportions of the haplogroups and the specific haplotypes of contributors present in a mixed sample.
Email:
brian@nichevision.com
Input data type:
Paired End FastQ
FastQ 1:
Choose File Sample_RX34_R1.fastq
FastQ 2:
Choose File Sample_RX34_R2.fastq
Seq error correction?
Use subset of reads?:
Max Total Reads
Max % Read
Show Advanced Options
Reset Options
Submit
Display Previous Results
MixtureAce
For Analysis of Mixed MPS Data

D.

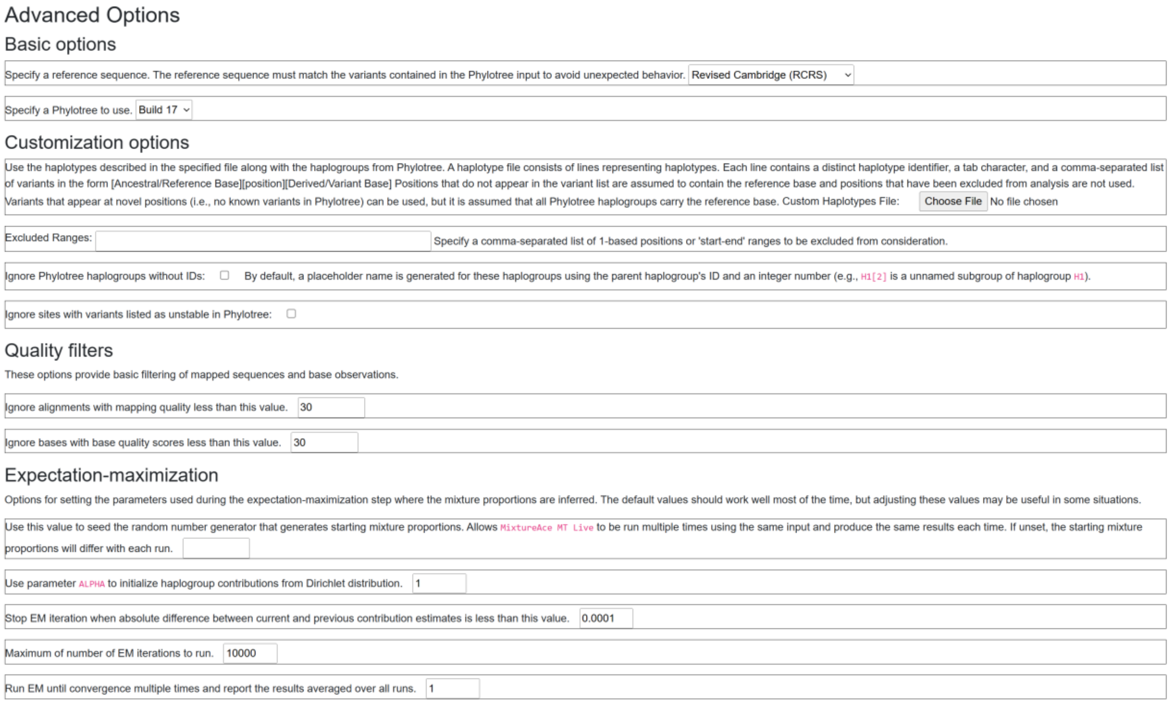
Advanced Options
Basic options
Specify a reference sequence. The reference sequence must match the variants contained in the Phylotree input to avoid unexpected behavior.
Revised Cambridge (RCRS)
Specify a Phylotree to use.
Build 17
Customization options
Use the haplotypes described in the specified file along with the haplogroups from Phylotree. A haplotype file consists of lines representing haplotypes. Each line contains a distinct haplotype identifier, a tab character, and a comma-separated list of variants in the form [Ancestral/Reference Base][position][Derived/Variant Base] Positions that do not appear in the variant list are assumed to contain the reference base and positions that have been excluded from analysis are not used. Variants that appear at novel positions (i.e., no known variants in Phylotree) can be used, but it is assumed that all Phylotree haplogroups carry the reference base. Custom Haplotypes File:
Choose File No file chosen
Excluded Ranges:
Specify a comma-separated list of 1-based positions or 'start-end' ranges to be excluded from consideration.
Ignore Phylotree haplogroups without IDs:
By default, a placeholder name is generated for these haplogroups using the parent haplogroup's ID and an integer number (e.g., H1[2] is a unnamed subgroup of haplogroup H1).
Ignore sites with variants listed as unstable in Phylotree:
Quality filters
These options provide basic filtering of mapped sequences and base observations.
Ignore alignments with mapping quality less than this value.
30
Ignore bases with base quality scores less than this value.
30
Expectation-maximization
Options for setting the parameters used during the expectation-maximization step where the mixture proportions are inferred. The default values should work well most of the time, but adjusting these values may be useful in some situations.
Use this value to seed the random number generator that generates starting mixture proportions. Allows MixtureAce MT Live to be run multiple times using the same input and produce the same results each time. If unset, the starting mixture proportions will differ with each run.
Use parameter ALPHA to initialize haplogroup contributions from Dirichlet distribution.
1
Stop EM iteration when absolute difference between current and previous contribution estimates is less than this value.
0.0001
Maximum of number of EM iterations to run.
10000
Run EM until convergence multiple times and report the results averaged over all runs.
1

**E.**

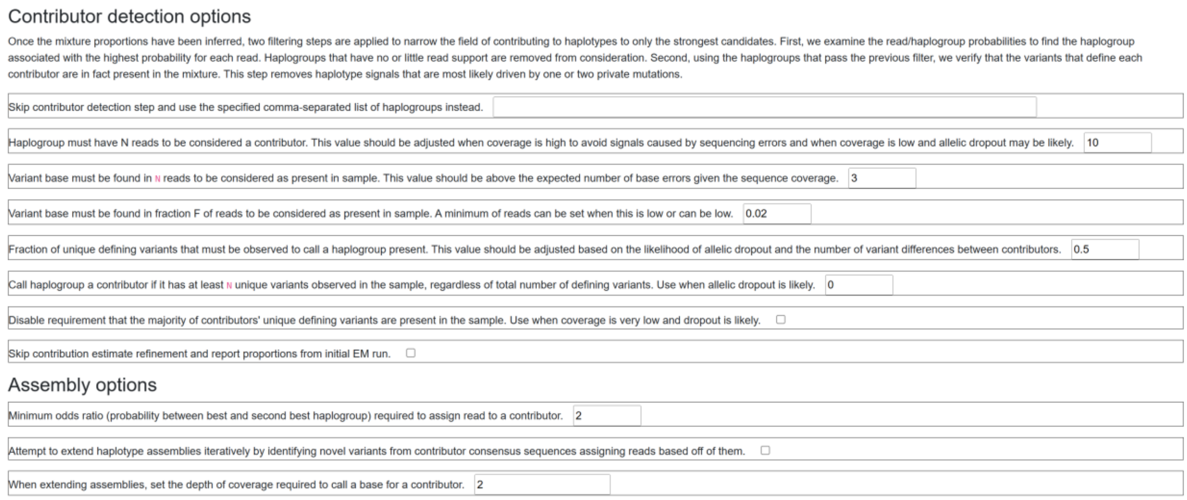
Contributor detection options
Once the mixture proportions have been inferred, two filtering steps are applied to narrow the field of contributing to haplotypes to only the strongest candidates. First, we examine the read/haplogroup probabilities to find the haplogroup associated with the highest probability for each read. Haplogroups that have no or little read support are removed from consideration. Second, using the haplogroups that pass the previous filter, we verify that the variants that define each contributor are in fact present in the mixture. This step removes haplotype signals that are most likely driven by one or two private mutations.
Skip contributor detection step and use the specified comma-separated list of haplogroups instead.
Haplogroup must have N reads to be considered a contributor. This value should be adjusted when coverage is high to avoid signals caused by sequencing errors and when coverage is low and allelic dropout may be likely.
10
Variant base must be found in N reads to be considered as present in sample. This value should be above the expected number of base errors given the sequence coverage.
3
Variant base must be found in fraction F of reads to be considered as present in sample. A minimum of reads can be set when this is low or can be low.
0.02
Fraction of unique defining variants that must be observed to call a haplogroup present. This value should be adjusted based on the likelihood of allelic dropout and the number of variant differences between contributors.
0.5
Call haplogroup a contributor if it has at least N unique variants observed in the sample, regardless of total number of defining variants. Use when allelic dropout is likely.
0
Disable requirement that the majority of contributors' unique defining variants are present in the sample. Use when coverage is very low and dropout is likely.
Skip contribution estimate refinement and report proportions from initial EM run.
Assembly options
Minimum odds ratio (probability between best and second best haplogroup) required to assign read to a contributor.
2
Attempt to extend haplotype assemblies iteratively by identifying novel variants from contributor consensus sequences assigning reads based off of them.
When extending assemblies, set the depth of coverage required to call a base for a contributor.
2

**F.**

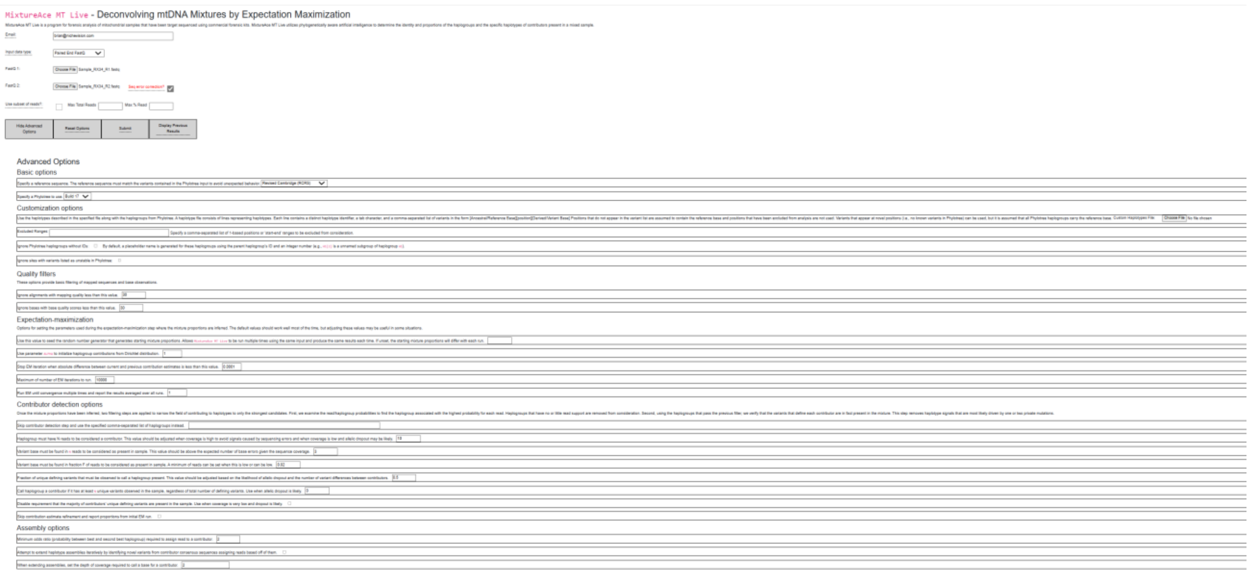
MixtureAce MT Live - Deconvolving mtDNA Mixtures by Expectation Maximization
Advanced Options
Basic options
Customization options
Quality filters
Expectation-maximization
Contributor detection options
Assembly options
